# Supplementary material for: Distinct pathways drive anterior hypoblast specification in the implanting human embryo
Source: Nat Cell Biol. 2024 Mar 5;26(3):353–65. doi: 10.1038/s41556-024-01367-1 (PMC10940163; doi:10.1038/s41556-024-01367-1)
Supplement: Supplementary file 1 — Captions for Supplementary Tables 1–8. [file 41556_2024_1367_MOESM1_ESM.pdf]

nature cell biology

Article https://doi.org/10.1038/s41556-024-01367-1

Distinct pathways drive anterior hypoblast specification in the implanting human embryo

In the format provided by the authors and unedited

Supplementary Table Descriptions

Supplementary Table 1 – Human Differential Gene Expression Analysis
Tables denoting differentially expressed genes identified by ROC analysis either between epiblast vs hypoblast vs trophectoderm/trophoblast, between trophoblast lineages, and across stages within each lineage for integrated human embryo scRNA-seq data.

Supplementary Table 2 – Cynomolgus Monkey Differential Gene Expression Analysis
Tables denoting differentially expressed genes identified by ROC analysis either between epiblast vs hypoblast vs trophectoderm/trophoblast vs extra-embryonic mesenchyme and across stages within each lineage for integrated cynomolgus monkey embryo scRNA-seq data.

Supplementary Table 3 – Mouse Differential Gene Expression Analysis
Tables denoting differentially expressed genes identified by ROC analysis either between epiblast vs visceral endoderm vs extraembryonic ectoderm and across stages within each lineage for integrated mouse embryo scRNA-seq data.

Supplementary Table 4 – Human Embryo Average Expression
Average expression following Seurat's SCTransform normalization for each stage across lineages in combined human sc-RNAseq data.

Supplementary Table 5 – Cynomolgus Monkey Embryo Average Expression
Average expression following Seurat's SCTransform normalization for each stage across lineages in combined cynomolgus monkey sc-RNAseq data.

Supplementary Table 6 – Mouse Embryo Average Expression
Average expression following Seurat's SCTransform normalization for each stage across lineages in combined mouse sc-RNAseq data.

Supplementary Table 7 – List of Wikipathways Gene Modules Used
List and annotation of gene lists used for module scoring

Supplementary Table 8 – Statistical Analysis Results
Coefficients and credible intervals for Bayesian analysis and exact p-values for all statistical tests presented.
