# Supplementary material for: Distinct pathways drive anterior hypoblast specification in the implanting human embryo
Source: Nat Cell Biol. 2024 Mar 5;26(3):353–65. doi: 10.1038/s41556-024-01367-1 (PMC10940163; doi:10.1038/s41556-024-01367-1)
Supplement: Supplementary file 2 — Reporting Summary [file 41556_2024_1367_MOESM2_ESM.pdf]

nature portfolio

Corresponding author(s): Magdalena Zernicka-Goetz

Last updated by author(s): Dec 1, 2023

Reporting Summary

Nature Portfolio wishes to improve the reproducibility of the work that we publish. This form provides structure for consistency and transparency in reporting. For further information on Nature Portfolio policies, see our Editorial Policies and the Editorial Policy Checklist.

Statistics

For all statistical analyses, confirm that the following items are present in the figure legend, table legend, main text, or Methods section.

n/a	Confirmed	
☐	☒	The exact sample size (*n*) for each experimental group/condition, given as a discrete number and unit of measurement
☐	☒	A statement on whether measurements were taken from distinct samples or whether the same sample was measured repeatedly
☐	☒	The statistical test(s) used AND whether they are one- or two-sided<br>*Only common tests should be described solely by name; describe more complex techniques in the Methods section.*
☒	☐	A description of all covariates tested
☐	☒	A description of any assumptions or corrections, such as tests of normality and adjustment for multiple comparisons
☐	☒	A full description of the statistical parameters including central tendency (e.g. means) or other basic estimates (e.g. regression coefficient) AND variation (e.g. standard deviation) or associated estimates of uncertainty (e.g. confidence intervals)
☐	☒	For null hypothesis testing, the test statistic (e.g. *F*, *t*, *r*) with confidence intervals, effect sizes, degrees of freedom and *P* value noted<br>*Give P values as exact values whenever suitable.*
☐	☒	For Bayesian analysis, information on the choice of priors and Markov chain Monte Carlo settings
☒	☐	For hierarchical and complex designs, identification of the appropriate level for tests and full reporting of outcomes
☐	☒	Estimates of effect sizes (e.g. Cohen's *d*, Pearson's *r*), indicating how they were calculated

Our web collection on statistics for biologists contains articles on many of the points above.

Software and code

Policy information about availability of computer code

Data collection	wget (1.21.2) was used to download publicly available data
Data analysis	Code to used to analyze these data is available at https://github.com/bweatherbee/PeriImplantation and combined sequencing atlas objects are available on Zenodo at https://doi.org/10.5281/zenodo.7689580.<br>List of software used -<br>command line tools - kallisto, kb-python, fastQC, cutadapt<br>R - R (4.1.2); Seurat (4.2.0), ggplot2 (3.3.6), Scillus (0.5.0), RColorBrewer(1.1-3), data.table (1.14.2), scmap (1.16.0), biomaRt (2.50.3), SingleCellExperiment (1.16.0), tidyverse (1.3.2), org.Hs.eg.db (3.14.0), rWikiPathways (1.14.0), brms (2.18.0)<br>Python - CellPhoneDB (2.1.7), python (3.8), matplotlib (3.3.0), anndata (0.7.5), scanpy (1.5.1), numpy (1.17.5)<br>GraphPad Prism (9.3)<br>Fiji (2.14.0)

For manuscripts utilizing custom algorithms or software that are central to the research but not yet described in published literature, software must be made available to editors and reviewers. We strongly encourage code deposition in a community repository (e.g. GitHub). See the Nature Portfolio guidelines for submitting code & software for further information.

Data

Policy information about availability of data

All manuscripts must include a data availability statement. This statement should provide the following information, where applicable:

- Accession codes, unique identifiers, or web links for publicly available datasets
- A description of any restrictions on data availability
- For clinical datasets or third party data, please ensure that the statement adheres to our policy

All raw data used here is previously published and publicly available.

For aligning sequencing data, GRCh38 (https://www.ncbi.nlm.nih.gov/assembly/GCF_000001405.26/), Genome assembly Macaca_fascicularis_5.0 (https://www.ncbi.nlm.nih.gov/datasets/genome/GCF_000364345.1/), and GRCm39 (https://www.ncbi.nlm.nih.gov/datasets/genome/GCF_000001635.27/) were used.

Human data
Molè et al., 2021: ArrayExpress E-MTAB-8060
Xiang et al., 2020: Gene Expression Omnibus GSE136447
Zhou et al., 2019: Gene Expression Omnibus GSE109555
Petropoulos et al., 2016: ArrayExpress E-MTAB-3929
Blakely et al., 2015: Gene Expression Omnibus GSE66507

Cynomolgus Monkey
Yang et al., 2021: Gene Expression Omnibus GSE148683
Ma et al., 2019: Gene Expression Omnibus GSE130114
Nakamura et al., 2016: Gene Expression Omnibus GSE74767

Mouse
Pijuan-Sala et al., 2019: ArrayExpress E-MTAB-6967
Mohammed et al., 2017: Gene Expression Omnibus GSE100597
Cheng et al., 2019: Gene Expression Omnibus GSE109071
Deng et al., 2014: Gene Expression Omnibus GSE45719

Human research participants

Policy information about studies involving human research participants and Sex and Gender in Research.

Reporting on sex and gender	We do not have access to prenatal genetic testing for the vast majority of embryos. Therefore, the composition of sex chromosomes of embryos cultured in the lab is largely unknown.
Population characteristics	According to the United Kingdom's Human Fertilisation and Embryology Act, which governs human embryo research, identifiable information of parents donating embryos to research is redacted. Therefore population characteristics of donating patients and their embryos is unknown.
Recruitment	Human embryos are donated by patients in the UK from collaborating IVF clinics under HFEA licence R0193. Patients undergoing IVF at CARE Fertility, Bourn Hall Fertility Clinic, Herts & Essex Fertility Clinic, and King’s Fertility was given the option of continued storage, disposal, or donation of embryos to research (including project specific information) or training at the end of their treatment. Patients were offered counseling, received no financial benefit, and could withdraw their participation at any time until the embryo had been used for research.<br><br>All information of patients is required to be redacted prior to donation to research. Therefore, potential biases based on recruitment is unknown. Please note this manuscript does not perform any experimentation on human embryos, rather we seek to provide a single example of an embryo cultured in vitro as a reference image for the natural post-implantation embryo.
Ethics oversight	Ethical oversight is provided both by the HFEA and the Human Biological Research Ethics Committee at the University of Cambridge. The recruitment of patients to donate human embryos to research follows the Human Fertilisation and Embryology Authority's guidelines. This includes the provision of project-specific information, the offering of counseling, and the ability to withdraw consent at any time until the embryos have been used. Stem cell work is approved by the UK Stem Cell Bank.

Note that full information on the approval of the study protocol must also be provided in the manuscript.

Field-specific reporting

Please select the one below that is the best fit for your research. If you are not sure, read the appropriate sections before making your selection.

☒ Life sciences ☐ Behavioural & social sciences ☐ Ecological, evolutionary & environmental sciences

Life sciences study design

All studies must disclose on these points even when the disclosure is negative.

Sample size	No tests were used to predetermine sample size. Sample sizes for experimentation was determined based on our previous experience with human embryos (Shahbazi et al., 2016, Mole et al., 2021) and human stem cells and 3D stem cell models (Shahbazi et al., 2017, Mackinlay et al., 2021, Weatherbee et al., 2023).
Data exclusions	For embryos assessed for normal development (Figures 2 and 3), only embryos that contained all three major lineages were included. For small molecule perturbation experiments, all embryos were included in all groups.<br>For single cell RNA-seq data, only those cells included for downstream analysis in the original publications were included in this study.
Replication	All experiments were performed at least twice, with a minimum of 5 embryos from 3 patients/crosses. Human embryo experiments were performed by 4 authors over time and mouse experiments by 2.
Randomization	Allocation was not performed randomly into groups for any experiments. For human and mouse embryo experiments, based on visual assessment of embryos, investigators attempted to ensure balanced distributions of blastocysts/implanting embryos assessed as expanded with nice inner cell masses versus embryos that appeared delayed or with visible cell death across experimental groups. For cell culture experiments, microwells with many cells or spheroids were all treated and quantified, and randomization/allocation of individuals would have been impossible.
Blinding	Investigators were not blinded to experimental groups in any of the human embryo, mouse embryo or stem cell experiments. It would not be feasible to blind the media changes with the addition of small molecules as we made media in-house.

Reporting for specific materials, systems and methods

We require information from authors about some types of materials, experimental systems and methods used in many studies. Here, indicate whether each material, system or method listed is relevant to your study. If you are not sure if a list item applies to your research, read the appropriate section before selecting a response.

Materials & experimental systems

n/a	Involved in the study	
☐	☒	Antibodies
☐	☒	Eukaryotic cell lines
☒	☐	Palaeontology and archaeology
☐	☒	Animals and other organisms
☒	☐	Clinical data
☒	☐	Dual use research of concern

Methods

n/a	Involved in the study	
☒	☐	ChIP-seq
☒	☐	Flow cytometry
☒	☐	MRI-based neuroimaging

Antibodies

Antibodies used	mouse monoclonal anti OCT3/4 (sc5279, Santa Cruz; clone C-10; 1:200 dilution), rat monoclonal anti SOX2 (14-19811-82, Thermo Fisher Scientific; clone Btjce; 1:500 dilution), goat polyclonal anti NANOG (AF1997 R&D Systems; 1:500 dilution), rabbit monoclonal anti GATA6 (5851, clone D61E4; Cell Signaling Technology; 1:2000 dilution), goat polyclonal anti GATA6 (AF1700, R&D Systems; 1:200 dilution), mouse anti monoclonal Cdx2 (MU392-UC, Biogenex; clone CDX2-88; 1:200 dilution), goat polyclonal anti CER1 (AF1075, R&D Systems; 1:250 dilution), rat monoclonal anti Cerebus1 (MAB1986, R&D Systems; clone 225807; 1:200 dilution), rabbit monoclonal anti Phospho-Smad1(Ser463/465)/Smad5(Ser463/465) /Smad9(Ser465/467) (13820T, Cell Signaling Technology; clone D5B10; 1:200 dilution), rabbit monoclonal anti Smad2.3 (8685T, Cell Signaling Technology; clone D7G7; 1:200 dilution), Rabbit monoclonal anti Cleaved Caspase 3 (9664, Cell Signaling Technology; clone 5A1E; 1:200 dilution), mouse monoclonal anti Podocalyxin (MAB1658, R&D Systems; clone 222328; 1:500 dilution), goat polyclonal anti Brachyury (AF2085, R&D Systems; 1:500 dilution), rat monoclonal anti GATA4 (14-9980-82, Thermo Fisher Scientific; clone eBioEvan; 1:500 dilution), goat polyclonal anti AP2-gamma (AF5059, R&D Systems; 1:500 dilution), goat polyclonal anti Otx2 (AF1979, R&D Systems; 1:1000 dilution), Alexa Flour 594 Phalloidin (A12381, Thermo Fisher Scientific; 1:500 dilution).
Validation	All antibodies are validated according to supplier's websites. Details of the validation statement, antibody profiles and relevant citations can be found on the manufacturer's website. In addition to that, all antibodies in this study showed expected staining patterns based on protein type (e.g. transcription factors in the nucleus, membrane-bound proteins at the membrane) in human embryonic stem cells.

Eukaryotic cell lines

Policy information about cell lines and Sex and Gender in Research

Cell line source(s)	UK Stem Cell Bank (Shef6), Prof Jennifer Nichols (Stem Cell Institute, University of Cambridge, UK) (mouse CD1 ESCs)
Authentication	The UK Stem Cell Bank validates deposited cell lines by STR analysis. mESCs were validated in-house by STR analysis.
Mycoplasma contamination	Cells were tested regularly for mycoplasma, and were negative.
Commonly misidentified lines (See ICLAC register)	No commonly misidentified lines were used in this study.

Animals and other research organisms

Policy information about studies involving animals; ARRIVE guidelines recommended for reporting animal research, and Sex and Gender in Research

Laboratory animals	CD1 wildtype males aged 6 to 45 weeks and CD1 wildtype females aged 6 to 18 weeks were used for this study. Mice were kept in an animal house in individually ventilated housing on 12:12 hour light-dark cycle with ad libitum access to food and water. Ambient temperature was maintained at 21-22ºC and humidity at 50%.
Wild animals	The study did not involve wild animals
Reporting on sex	Sex was not considered in this study as embryos were recovered from the mother and used for experimentation at the 8-cell stage. Genotyping was not performed.
Field-collected samples	Field-collected samples were not used in this study.
Ethics oversight	Mice were kept in an animal house on 12:12 hour light dark cycle with ad libitum access to food and water. Experiments with mice are regulated by the Animals (Scientific Procedures) Act 1986 Amendment Regulations 2012 and carried out following ethical review by the University of Cambridge Animal Welfare and Ethical Review Body (AWERB). Experiments were approved by the Home Office under Licenses 70/8864 and PP3370287. Animals were inspected daily and those showing health concerns were culled by cervical dislocation.

Note that full information on the approval of the study protocol must also be provided in the manuscript.
